# From tradition to innovation: conventional and deep learning frameworks in genome annotation

**DOI:** 10.1093/bib/bbae138

**Published:** 2024-04-04

**Authors:** Zhaojia Chen, Noor ul Ain, Qian Zhao, Xingtan Zhang

**Affiliations:** National Key Laboratory for Tropical Crop Breeding, Shenzhen Branch, Guangdong Laboratory for Lingnan Modern Agriculture, Genome Analysis Laboratory of the Ministry of Agriculture, Agricultural Genomics Institute at Shenzhen, Chinese Academy of Agricultural Sciences, Shenzhen, Guangzhou 518120, China; College of Biomedical Engineering, Taiyuan University of Technology, Jinzhong 030600, China; National Key Laboratory for Tropical Crop Breeding, Shenzhen Branch, Guangdong Laboratory for Lingnan Modern Agriculture, Genome Analysis Laboratory of the Ministry of Agriculture, Agricultural Genomics Institute at Shenzhen, Chinese Academy of Agricultural Sciences, Shenzhen, Guangzhou 518120, China; State Key Laboratory for Ecological Pest Control of Fujian/Taiwan Crops and College of Life Science, Fujian Agriculture and Forestry University, Fuzhou, 350002, China; National Key Laboratory for Tropical Crop Breeding, Shenzhen Branch, Guangdong Laboratory for Lingnan Modern Agriculture, Genome Analysis Laboratory of the Ministry of Agriculture, Agricultural Genomics Institute at Shenzhen, Chinese Academy of Agricultural Sciences, Shenzhen, Guangzhou 518120, China

**Keywords:** genome annotation, genome sequence, bioinformatic, RNA-Seq technology, deep learning

## Abstract

Following the milestone success of the Human Genome Project, the ‘Encyclopedia of DNA Elements (ENCODE)’ initiative was launched in 2003 to unearth information about the numerous functional elements within the genome. This endeavor coincided with the emergence of numerous novel technologies, accompanied by the provision of vast amounts of whole-genome sequences, high-throughput data such as ChIP-Seq and RNA-Seq. Extracting biologically meaningful information from this massive dataset has become a critical aspect of many recent studies, particularly in annotating and predicting the functions of unknown genes. The core idea behind genome annotation is to identify genes and various functional elements within the genome sequence and infer their biological functions. Traditional wet-lab experimental methods still rely on extensive efforts for functional verification. However, early bioinformatics algorithms and software primarily employed shallow learning techniques; thus, the ability to characterize data and features learning was limited. With the widespread adoption of RNA-Seq technology, scientists from the biological community began to harness the potential of machine learning and deep learning approaches for gene structure prediction and functional annotation. In this context, we reviewed both conventional methods and contemporary deep learning frameworks, and highlighted novel perspectives on the challenges arising during annotation underscoring the dynamic nature of this evolving scientific landscape.

## INTRODUCTION

The successful completion of the Human Genome Project (HGP) has propelled genome sequencing efforts in various model organisms, including *Escherichia coli*, yeast, Drosophila and mice. For instance, the complete sequencing data of the *E. coli* genome was obtained in 1997, and in the year 2000, the first plant genome, *Arabidopsis thaliana*, was entirely sequenced. Nevertheless, the genome sequences of various organisms obtained through sequencing are nebulous in unraveling the complete molecular processes of life. With advancements in the field of sequencing, it is of paramount importance to decipher the hidden information within DNA sequences. As emphasized by Collins, the chief scientist of the HGP, the next phase of genome research involves elucidating the structure and function of genomes [1] and establishing connections between genomics and biology. Therefore, in 2003, scientists collectively launched the Encyclopedia of DNA Elements (ENCODE) project with the aim of mining and deciphering numerous functional elements within the human genome [2].

With the rapid advancement of Next-Generation Sequencing technologies, the ENCODE website has been updated to the fifth edition, providing researchers with functional genomics and omics data including ChIP-Seq and RNA-Seq experiment reads. This update has opened new doors of opportunities for genomics research, potentially providing access to billions of genomic coordinates and other related data. Typically, after sequencing the genome of a biological organism, the first step involves the correction and assembly of sequenced fragments to generate the base sequence of the genome. With the genome sequence in hand, a world of possibilities unfolds, enabling us to meticulously delve into systematic analysis unravelling the functions of all genes, their interactions, decoding the regulation of genes during an organism’s growth and development, and to elucidate the evolutionary patterns of genomes.

Genome annotation, accomplished through the application of bioinformatics methods and tools, entails the identification of genes and various functional elements, including coding genes [3], non-coding RNAs [4]^,^, repetitive sequences such as transposons, and regulatory elements, within a genome. Traditional approaches for genome annotation, such as hybridization-based techniques [5] or experimental methods [3, 4], heavily rely on human knowledge and expertise, often with limited throughput and high costs. In addition to these, various bioinformatics software tools have been developed for gene identification and annotation, such as Blast2GO [6], InterProScan [7] and GeneMark [8], among others. However, these methods and softwares primarily constitute shallow learning approaches, with limited capabilities to handle high-throughput data, posing challenges in terms of cost and technical accessibility for researchers without backgrounds in biology and medicine. Furthermore, according to a report by McKinsey [9], it is estimated that by 2025, the scientific databanks will accumulate one billion human genomes, presenting a significant challenge in the field of bioinformatics for the analysis of large-scale omics data.

Over the past decade, deep learning has achieved significant success in fields such as computer vision, speech recognition and natural language processing. The development of machine learning and deep learning has opened new avenues for genome analysis. Many researchers use machine learning techniques to identify patterns in genomic datasets, processing and analyzing data from a variety of biomolecular levels through statistical analysis and computational modeling. Deep learning, in particular, employs multi-layered nonlinear functions to abstract data, extract representative features from large-scale datasets and make more accurate predictions regarding DNA fragments; however, deep learning models are more capable and flexible. With appropriate training data and models, deep learning can automatically learn features and rules with minimal human participation. Several deep learning methods have already been developed for the identification of various genomic elements, such as exons [10], promoters [11] and so on. This article aims to provide a concise overview of the application of deep learning and machine learning in genome annotation research and highlight potential directions for future development.

To comprehend the current topic, we briefly introduce conventional algorithms in genome annotation. And we subsequently review methods and approaches for the identification and functional prediction of DNA segments with biological significance using deep learning. Finally, we summarize the current challenges and potential research directions.

## TRADITIONAL APPROACHES TO GENOME ANNOTATION

Genome annotation, often referred to as the high-throughput annotation of the biological functions of all genes in a genome, utilizes rigorous bioinformatics methods and tools to search and define genomic elements using various approaches, including ab initio prediction, homology-based methods and structural definitions [12, 13]. In general, a complete and effective genome annotation process mainly includes repeat sequence annotation, gene structure prediction, gene functional annotation, variant analysis and the identification of regulatory elements. Organisms are divided into prokaryotes and eukaryotes. The basic ideas and methods of genome annotation are similar. However, due to the large genome of eukaryotes and the large number of repeats, the genome annotation process is more complicated, so this section mainly introduces the genome annotation process of eukaryotes.

Repetitive sequences, defined as identical or symmetric DNA sequences occurring in genomes [14], influence processes such as evolution, gene expression, and transcriptional regulation [15–17]. The recognition of repetitive sequences is the fundamental task in genome annotation and is a prerequisite for subsequent gene prediction. Traditional methods for identifying repetitive sequences fall into three categories: homology-based, structure-based and *ab initio* prediction [18–20]. Repeatmasker software, widely used for repetitive sequence annotation, employs homology-based identification by conducting similarity searches against repetitive sequence libraries (RepBase and Dfam). MASiVE [21], on the other hand, utilizes heuristic algorithms and structural features to analyze species-specific Long Terminal Repeats (LTR) transposons in plants. The *ab initio* prediction method, which does not rely on repetitive structures or known sequence similarity information, is particularly useful for novel sequences and is often more flexible than the aforementioned two methods [18, 22]. However, because it mainly analyzes the sequence structure and characteristics based on mathematical models, it is susceptible to algorithm selection and parameter settings, and has high computational complexity when processing large datasets, and may even produce false positives. Therefore, conventional methods are difficult to obtain satisfactory results in terms of accuracy and dimensions for diverse genomes.

Gene structure prediction represents one of the most critical aspects of genome annotation, aiming to identify coding regions, exons, introns and other functional elements within a genome. Traditional prediction methods combine structural features of genes with evidence from gene expression and homologous protein information from closely related species [23]. The traditional prediction method is based on the structural characteristics of genes while integrating gene expression and homologous protein evidence from related species [23]. Taking the Maker [23] as an example, the Semi-HMM-based Nucleic Acid Parser model is first trained using protein sequences, Expressed Sequence Tag sequences, and homologous proteins from closely related species. A specialized configuration file is established to control the operation of the process, and the Marker is iteratively employed based on the output results to obtain the optimal result. It is worth mentioning that the parameters of the configuration file need to be manually configured, resulting in low-level of automation performance. Moreover, the reliability of analysis requires a large number of input samples. However, high-dimensional data increase the complexity of analysis, requiring more powerful computing resources and more data preprocessing work. In addition, methods based on structural features such as Genscan [24], GlimmerHMM [25], Augustus [26] and Apollo [27] focus on specific base structures, and the corresponding elements are generally identified by specific nucleotide sequences (e.g. open reading frames, donor sites, TATA boxes). With the continuous development of sequencing technologies and approaches, novel genomic and transcriptomics data are continually emerging, potentially revealing new genes or critical structural information and identifying genes expressed in specific tissues or physiological states [28]. Consequently, mainstream genome annotation tools commonly integrate transcriptomic data to aid in genome annotation [29].

After obtaining gene structure annotation information, the crucial step is to proceed with functional annotation, including predicted domains, protein functions and biological pathways. Typically, functional annotation of predicted coding genes is achieved through sequence similarity searches, primarily utilizing Basic Local Alignment Search Tool alignment methods, by comparing proteins to various functional databases [NR, Swiss-Prot, Gene Ontology (GO), KOG, Kyoto Encyclopedia of Genes and Genomes (KEGG)] to obtain information regarding gene function. As genomics and bioinformatics advance, an increasing number of species’ genome sequences are being collected, necessitating the continuous exploration of new genome functions and biological processes. For some newly discovered gene sequences, especially those lacking annotation information, experimental methods are usually required to determine gene function definitively.

Furthermore, traditional methods tend to yield high accuracy in predicting genes with highly conserved structures but may fall short in predicting genes with significant structural variations (SVs) [30]. Genome variations between individuals are typically manifested as single nucleotide variations (SNVs), insertions, deletions and SVs. For second-generation sequencing, such as Illumina, with shorter read lengths, predicting complex SVs, particularly those with unknown structures, poses challenges, and conventional methods based on read depth [31], local assembly [32] or other methods [33] may be insufficient [34]. With the rapid development of long-read sequencing technologies, such as Pacific Biosciences [35] and Oxford Nanopore Technologies [36], information containing complete complex SVs is becoming available. Nevertheless, advancements in bioinformatics and computational methods are still required to handle highly repetitive sequences, high sequencing error rates and long read lengths [37, 38]. Moreover, SVs in the genome often occur in highly repetitive regions, making it challenging to distinguish SV intervals from background noise.

Of greater importance, eukaryotic genome sequences encompass DNA methylation sites, promoters, enhancers and numerous regulatory elements. Traditional methods, which rely on sequence logic based on consensus sequences and positional weight matrices, offer limited information and accuracy in predicting the activation levels and behaviors of regulatory elements [39]. Consequently, research into gene regulation mechanisms encounters significant limitations. In summary, genome annotation is an ongoing and evolving process that necessitates the incorporation of the latest technologies and methods to continually enhance annotation accuracy and reliability.

## APPLICATION OF DEEP LEARNING IN GENOME ANNOTATION

Deep learning, a branch of machine learning, is considered a significant step towards artificial intelligence. The essence is to learn the intrinsic laws and latent representation of massive sample data by constructing multiple hidden layers. In the field of bioinformatics, deep learning techniques have gradually found applications in genome annotation research [40, 41]. In this section, we will introduce the technical methods and application cases of deep learning in genome annotation.

### The potent tool for genome annotation—deep learning

**Figure 1:**
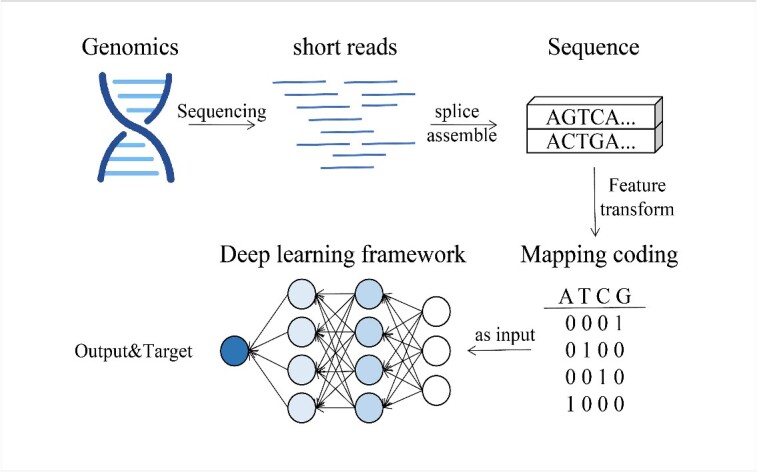
Deep learning workflow in genome annotation. The input data of the deep learning model is raw sequence data, obtained from short reads obtained by genome sequencing after sequence splicing and assembly. Before inputting into the deep learning model, it first undergoes feature encoding and then input into a deep learning network constructed by multiple hidden layers. The deep learning network extracts potential features from the input data through multiple hidden layers for subsequent classification and prediction of genomic components.

Deep learning is essentially a Deep Neural Network (DNN) architecture consisting of an input layer, multiple hidden layers and an output layer. Firstly, raw sequencing data are subjected to feature encoding (e.g. one-hot encoding [42], word embeddings [43], k-mer Counting [44]) and are mapped into the input representation of the deep learning model. Unlike traditional genome annotation methods, deep learning models can model nonlinear patterns and adaptively learn feature representations from raw data without the need for manual feature design. This is achieved by embedding the computation of features directly into the machine learning model, resulting in an end-to-end model, as depicted in Figure 1.

The most significant capability of deep learning is representation learning, achieved through three key steps: layer-by-layer processing, feature transformation and increasing complexity. The raw data in genome annotation comprise various types and numerous features, originating from different data sources or datasets, hence possessing high dimensionality and heterogeneity. Deep learning extracts latent features from high-dimensional heterogeneous data, facilitating subsequent prediction and classification tasks. In the face of growing data volumes, deep learning models can enhance the width of expressive power by increasing network depth and width, and they accelerate the training process through parallel computation. With these characteristics, deep learning becomes a powerful tool for genome annotation with broad application prospects.

Deep learning models for sequence processing include Convolutional Neural Networks (CNN), Recurrent Neural Networks (RNN); furthermore, CNN is the primary algorithm.

**Figure 2:**
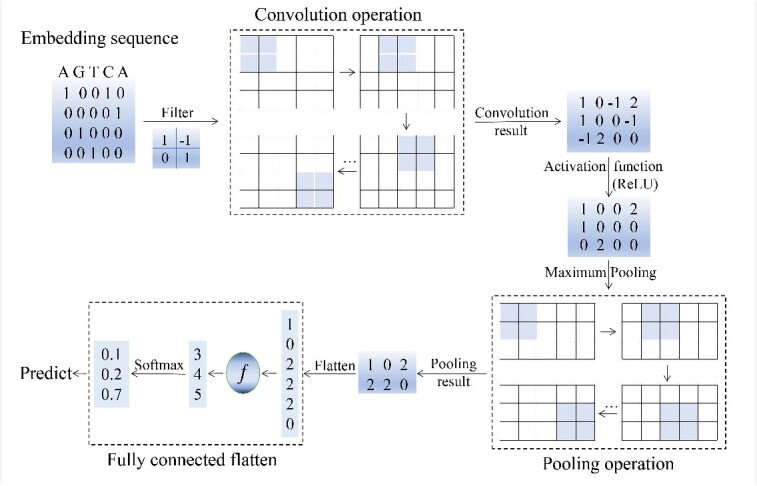
Structure diagram of convolutional neural network. The basic convolutional neural network consists of five parts: input layer, ReLU layer, pooling layer, fully connected layer and output layer. The input layer is an embedding sequence, and the convolutional layer performs convolution operations on local data through a filter. The activation layer maps the convolutional calculation results nonlinearly (usually through the activation function ReLU). The pooling layer is sandwiched between continuous convolutional layers to compress the amount of data and parameters. The fully connected layer integrates feature representations together, and finally predicts through the output layer.

CNN is a type of multi-layer perceptron akin to artificial neural networks and comprises convolutional layers, pooling layers and fully connected layers. It was pioneered by renowned computer scientist Yann LeCun [45]. The core of CNN is its convolutional and pooling layers. As illustrated in Figure 2, the convolutional layer detects features at different positions by sliding convolution kernels across the sequence, used to identify critical patterns within the sequence [46]. Importantly, the parameters of these convolution kernels are learned automatically during model training. In convolutional operations, parameters are shared among all positions in the feature map or feature vector, substantially reducing the number of parameters that need to be learned within the network. While convolution operations in neural networks essentially represent linear data processing, not all situations can be simplified as linear processing. The genome contains billions of base pairs and a large number of functional elements, and their interactions and regulatory relationships are nonlinear. Especially for the annotation of three-dimensional genomics and SVs, nonlinear modeling is needed to better understand and explain. This is where activation functions come into play to address the non-linearity in the model [47]. Common activation functions in CNN include ReLU, sigmoid and tanh, which are used for nonlinear transformation to enable the extracted features to represent complex functions.

Pooling layers undergoes repetitive subsampling similar to the convolutional layer, max-pooling selects the maximum value in each region, while average pooling computes the average value. These layers serve to reduce dimensionality and extract the most critical features.

Following the convolutional and pooling layers, neural networks often include one or more fully connected layers. These layers serve to flatten the highly abstracted features processed through multiple convolutional and pooling steps into one-dimensional vectors, reducing the impact of feature positions on classification. For instance, motifs in sequences may exhibit similar features at different positions, which could lead to varying classification results. The role of fully connected layers, in this context, is akin to aggregating all regions with similar features into a same value, greatly enhancing the model’s robustness. Consequently, CNNs are highly useful when dealing with scenarios that require recognition or capturing spatially invariant patterns [48].

**Figure 3:**
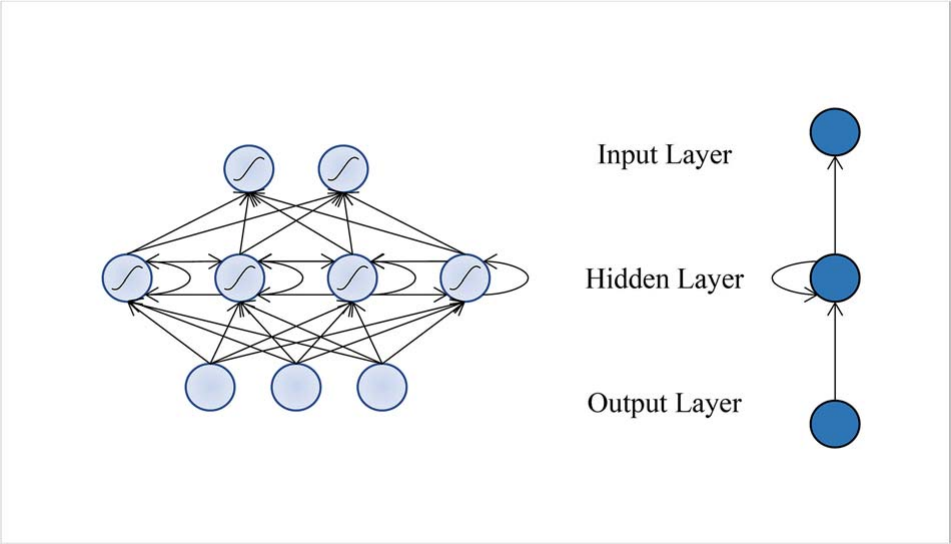
Structure diagram of the Recurrent Neural Network. The basic Recurrent Neural Network consists of three parts: input layer, hidden layer and output layer. In the hidden layer, Recurrent Neural Network stores and remembers previous information, and then inputs it into the current calculated hidden layer unit.

Another variant of DNNs is RNN [49, 50]. The traditional neural network model is fully connected between the layers, which will ignore the context information of the sequence. RNN introduces recurrent connections to save the information of each layer, and synchronously updates the information of the hidden state to the new network layer. This mechanism ensures that the information at any given moment is determined not only by the input layer at that specific time but is also influenced by the preceding process’s output, as illustrated in Figure 3. Since the sequences themselves may carry continuous information, RNN can share weights across time and extract the temporal and semantic information in the data, Therefore, RNN has unique specifications in identifying the patterns with in long and sequential data.

However, traditional RNNs suffer from problems like vanishing gradients and exploding gradients, making it challenging to capture long-range dependencies [51, 52]. Fortunately, the introduction of models like Long Short-Term Memory (LSTM) [53] and Bidirectional RNNs [54] have addressed these issues. LSTM models incorporate gate units to control information flow, allowing dynamic changes in states across different nodes. The bidirectional RNN considers the context relationship. For each training sequence, it connects two recurrent networks forward and backward, and both networks connect an output layer. Importantly, there is no interconnection of information flow between the forward and backward networks, ensuring a non-cyclic unfolded graph. Overall, the RNN has significant advantages in processing genomic sequence data, which can better capture the features of the sequence and more accurately predict important biological features such as gene location and functional regions.

In addition to CNNs, RNNs and their corresponding variants, a variety of deep learning models such as attention mechanism-based neural networks (such as Transformer [55]), graph convolutional network [56] and autoencoder [57] have been widely used in genome annotation. The choice of deep learning models is not independent of each other, and appropriate models can be selected according to different needs and data types to improve the accuracy and interpretability of genome annotation. The quality and scale of the training set significantly affect the feature learning process in deep learning. Before inputting into the model, data preprocessing is necessary to address issues such as handling erroneous labels, missing values, and outliers. Furthermore, the training set should encompass samples from all important categories or scenarios to avoid biases in the model.

Preventing overfitting is a crucial concern when training models. Firstly, selecting an appropriate model complexity is essential. If the model is overly complex, it may overfit the training data, leading to poor performance on unseen data. Therefore, striking a balance between model complexity and performance is necessary. Secondly, regularization techniques are commonly employed to prevent overfitting. By adding penalty terms [58] to the loss function, regularization techniques can constrain the size of model parameters, thereby reducing the risk of overfitting. Additionally, cross-validation [59] is an effective method for assessing model performance and selecting optimal hyperparameters. By dividing the dataset into training and validation sets and conducting multiple training and evaluation iterations, a better estimation of the model’s generalization ability can be obtained. Lastly, early stopping [60] is another effective method for preventing overfitting. Monitoring the model’s performance on the validation set during training and stopping the training process once the performance no longer improves helps prevent overfitting.

### Applications in genome annotation

In this section, we briefly review some of the genome annotation problems addressed using deep learning methods and discuss how deep learning models extend their applicability in various genomics areas, as shown in Table 1.

**Table 1:** Genomic tools/algorithm based on deep learning architecture for Genome Annotation.

Tools	ML&DL model	Application	Input	Year	References
nLLCPN+LCPNB	MLPs	The first attempt to propose Hierarchical Classifiers of TEs.	REPBASE1, PGSB2	2017	54
SClassifyTE	optimized SVM with RBF kernel	(1) Higher accuracy; (2) lack of training data results in poor classification performance at super family level.	PGSB, REPBASE	2021	55
DeepTE	CNN	(1) A tree structured classification process to classify TEs into super families and orders; (2) detected domains inside TEs to correct false classification; (3) K-mer-based computational approaches are time-consuming.	PGSB, REPBASE	2020	58
Inpactor2	CNN	(1) The first NN-based tool to detect LTR-retrotransposons de novo; (2) can be run using CPUs + GPUs, speeding up the execution time up to 7 times.	REPBASE, PGSB, REPETDB	2023	60
None	DNN	(1) Identification of protein coding region by Deep Belief Network model; (2) overcome the barriers of traditional protein coding region identification techniques.	BG570, HMR195, GEN_x005fSCAN65	2020	63
SpliceAI	CNN	(1) Accurately predicts splice junctions from an arbitrary pre-mRNA transcript sequence and noncoding genetic variants that cause cryptic splicing; (2) bio interpretability is low, and the understanding of how noncoding genomic mutations cause human disease is still far from complete.	GENCODE-annotated pre-mRNA transcript sequences	2019	64
Bidirectional LSTM-RNNs	RNN	Identification and prediction of splice sites of eukaryotic DNA.	*Cryptosporidium parvum*	2022	88
Gene2vec	word2vec (ML) + DNN	Utilizes transcriptome-wide gene co-expression to generate a distributed representation of genes.	Gene expression data, gene type data, gene–gene interaction data, tissue-specific gene expression data	2019	66
GeneWalk	DeepWalk (ML)	(1) Utilizes network representation learning to embed genes; (2) introduces bias towards genes that have been studied.	Gene expression data, Dynamical Reasoning Assembler (INDRA) statements	2021	67
DeepGMAP	CNN(DL)	(1) Efficiently identifies patterns in regulatory DNA sequences; (2) can process information from both forward and reverse sequences; (3) comparison with other models and addressing runtime memory issues are necessary.	Alignment files, containing data from chromatin accessibility assays and chromatin immunoprecipitation- sequencing experiments from the ENCODE project, and regions enriched with reads were determined as peaks by MACS2 peak caller	2018	68
deepNF	AE(DL) + SVM(ML)	(1) Using autoencoders to construct compact low-dimensional protein feature representations from various types of networks; (2) accurate annotation of proteins with functional labels.	The protein–protein interaction networks of both human and yeast contained in the STRING database	2018	69
DeepGO	CNN	(1) Combining two forms of multi-layer neural network-based representation learning to acquire useful features for predicting protein function; (2) require a large amount of training data and consumes significant computational resources.	The amino acid (AA) sequence of a protein	2018	70
LncADeep	DNN	LncADeep demonstrates competitive time efficiency compared to state-of-the-art tools in lncRNA identification, and it stands out as the fastest tool for large-scale prediction of lncRNA–protein interactions.	RefSeq, GENCODE	2018	71
HMPI	CNN	Simultaneously simulating structural profiles of promoters and the original sequences of promoters for comprehensive promoter identification, and successfully applied to human, plant, and *Escherichia coli K-12 strain* datasets.	Eukaryotic Promoter Database (EPD), PlantProm DB	2022	7
BERT-DNA	CNN	Using pre-trained BERT model to extract features enables efficient identification of DNA enhancers from sequence information.	The chromatin state information of nine cell lines, including H1ES, K562, GM12878, HepG2, HUVEC, HSMM, NHLF, NHEK and HMEC	2021	75
DeepArk	CNN	(1) Predicting the regulatory activity of cis-elements from the DNA sequences and genomic variants of four widely studied model organisms. (2) Affected by data limitations and contextual complexity, optimization of CNN architectures and sequence lengths is required.	*Caenorhabditis elegans*, *D. rerio*, *D. melanogaster* and *M. musculus*	2021	76
DeepVariant	CNN	(1) Transforming the problem of distinguishing between different mutation types and lengths into an image classification task; (2) efficiently identifying mutation sites using CNN.	Next-generation sequencing (NGS) reads and reference genome	2018	87
Basset	CNN	(1) Predicting pathogenic single nucleotide polymorphisms (SNPs); (2) lack the ability for de novo annotation of large genomes.	DNA sequence information derived from DNase I hypersensitive sites (DHS)	2016	78
MuRaL	CNN	(1) Learning mutation information from proximal and distal sequence environments to generate high-quality mutation rate maps for humans and multiple species; (2) uneven coverage of genomic regions; (3) limited scope for other mutation types.	The DNA sequences of four representative species—*H. sapiens*, *M. mulatta*, *A. thaliana* and *D. melanogaster*	2022	79
SVision	CNN	Detect and characterize complex structural variants (CSVs) from long-read sequencing data.	Long-read sequencing data	2022	80

ML: machine learning, DL: deep learning, MLP: multi-layer perceptron, SVM: support vector machine, CNN: Convolutional Neural Network, DNN: Deep Neural Network, AE: autoencoder.

#### Identification and classification of transposable elements

Transposable elements (TEs), also known as transposons, are the most common repetitive sequences [61] and are widely present in eukaryotic genomes. TEs show great diversity across species and individuals, with in genomes. Moreover, owning to the recurrent process of genome evolution, TEs undergo modifications at large levels [62]. This ongoing and incessant unceasing evolution complicates the task of annotating TEs, making it a tough nut to crack. Traditional bioinformatics software, relying on de novo, structural, comparative genomics and homology-based methods, often suffer from high false-positive rates [63].

While some studies have employed machine learning methods for TE identification [64, 65], recent advancements suggest that deep learning techniques can yield better results [66, 67]. Yan et al. developed the DeepTE tool [68], which transforms sequence data into two-dimensional vectors based on k-mer counts. DeepTE utilizes CNN to train on datasets from RepBase and PGSB databases. It employs a stacked architecture with eight models to classify TEs into super families and orders. DeepTE also employs conservative thresholds to correct misclassifications, achieving TE classification into 15–24 super families for plants, metazoans and fungi. However, k-mer-based computational approaches are time-consuming [69] and can negatively impact neural network training. Consequently, Orozco-Arias et al. developed Inpactor2 [70], which can create an LTR-retrotransposon reference library in a short time frame. By combining structural methods with deep learning and leveraging multi-core and GPU architectures, Inpactor2 achieves the best testing accuracy in just five minutes for the rice genome.

In summary, deep learning techniques have made significant breakthroughs in the field of TE annotation. Despite of offering higher identification accuracy, they are able to handle diversity across different species and individuals more effectively, addressing the challenges posed by TE evolution and variation. These tools not only enhance our understanding of TEs in the genome but also provide vital resources for further research into genome evolution, function and regulation.

#### Protein-coding genes

The majority of eukaryotic gene coding regions are discontinuous, with exons and introns alternatively connected [5], and without fixed positions. Moreover, eukaryotic genomes exhibit extensive diversity in length, composition and structure, including variations such as repeat sequences and splice isoforms, making it difficult for traditional machine learning methods to fully capture coding region features [71, 72].

Deep learning-based methods map the nucleotide characters of gene sequences into feature spaces, extracting abstract features from numeric sequences to effectively utilize global information. Recently, approaches using DNNs, such as Deep Belief Networks, have been proposed for exon-intron classification [10]. Qingyu et al. first processed the DNA sequences of eukaryotes using short-time Fourier transform, transforming complex DNA strings into numerical sequences. After using Random Forests for feature selection, the extracted feature set is used as the discriminant variable, and the known coding discriminant result is used as the discriminant target to construct a deep belief network model. The comparison on three standard test datasets show that the accuracy and specificity of the deep belief network model are significantly better than those of the logistic regression model and the Bayesian discriminant model.

Alternative splicing is a crucial step in protein-coding gene transcription, and accurate identification of splice sites is essential for understanding and analyzing protein coding. SpliceAI [73], which does not rely on predefined features, predicts splice function solely based on pre-mRNA transcripts as an input. It constructs a 32-layer DNN that predicts pre-mRNA splice sites by evaluating 10,000 nucleotides of context sequences, directly identifying splice sites from primary sequences. However, as a black-box model, deep learning has low bio interpretability, so the understanding of how mutations in non-coding genes cause human disease is far from complete. Singh et al. combined bidirectional LSTM and recursive neural network models for identifying and predicting splice sites in eukaryotic DNA, facilitating exon recognition [74].

In conclusion, deep learning has made progress in identifying and analyzing protein-coding regions. With the continuous emergence of genomic data, deep learning will have more opportunities in predicting gene start and stop positions, splice sites, exons, introns and other aspects.

#### Functional annotation

Existing gene functional analogies are discrete and primarily generated through manual processes [43]. The widespread application of high-throughput experiments has resulted in extensive molecular interaction networks, providing rich sources of information for gene and protein functional annotation. DNNs can learn from various types of biological data, inferring interactions between genes and biological functions followed by training the model. Gene2vec [43] utilizes the Skip-gram algorithm from the Word2Vec neural network model to transform gene expression data into text, generating distributed representations of genes based on gene co-expression network of the transcriptome, thus predicting functions of unknown genes. This method has shown promising results in gene annotation tasks across multiple species.

The most commonly used softwares for gene functional annotation are GO and the KEGG. However, these methods do not provide specific functional information for genes, meaning that the functions of annotated genes may not be related to the so-called biological processes, molecular functions in the network. Thus, finding the most relevant functions for genes in specific experimental contexts is a challenge in gene functional annotation. The Churchman lab developed the Genewalk method [75], which extends GO annotation by learning network representations to identify the most important genes and their related functions in gene lists, facilitating the identification of key genes and downstream experimental breakthroughs. Because the study used the INDRA and Pathway Commons knowledge bases to assemble the GeneWalk network, there are certain limitations when dealing with more distant species, such as those more closely related to humans.

Deep learning has been applied to predict relationships between genes and phenotypes across multiple species. A typical example is DeepGMAP [76], a deep learning-based genotype–phenotype mapping platform. It trains various neural network architectures on epigenomic data and incorporates ideas from graph embedding and attention mechanisms to predict associations between genes and phenotypes. The model can process both positive and negative sequence information, but comparing with other models and addressing runtime memory issues are necessary.

Moreover, researchers have utilized deep learning methods to predict the functions of non-coding RNAs [77] and protein–protein interactions [78, 79], respectively. Long non-coding RNAs (lncRNAs) share many similarities with mRNAs, such as transcript length and splicing structure, making lncRNA annotation challenging. Yang et al. developed LncADeep [77], the first tool to identify and infer the functions of lncRNAs. The model integrates intrinsic and homologous features of sequences and employs deep belief networks to discriminate transcripts, incorporating annotations from KEGG and Reactome, among others, for functional annotation.

#### Regulatory elements

In addition to protein-coding regions, the genome contains crucial regulatory elements that determine when, where and how genes are expressed. A deep understanding of regulatory elements is essential for elucidating the mechanisms of life, the causes of human diseases and conservation patterns among species [80, 81]. In recent years, DNNs have demonstrated outstanding performance in the identification of promoters, enhancers and transcription factors.

Wang et al. proposed a hybrid model, HMPI [11], for promoter recognition, which combines fully connected networks and DenseNet-based Deep Structural Profile Networks, achieving excellent performance on datasets for plants, humans, and *E. coli*.

Enhancers, which increase the transcriptional activity of promoters, are critical regulatory elements. Researchers have recently improved enhancer prediction by processing DNA sequences with pre-trained Bidirectional Encoder Representations from Transformers [82]. This approach combines bidirectional encoding with CNNs to capture interpretable features, resulting in significant enhancements in enhancer prediction performance. Identifying transcription factor binding sites is equally crucial for understanding transcriptional regulation. DeepArk [83], a deep learning model for studying cis-regulatory activity across four extensively researched species, accurately predicts thousands of different regulatory features, including chromatin states, histone marks and transcription factors. Despite predicting thousands of regulatory features, DeepArk may still miss modeling certain regulatory features due to data limitations. For example, it may not cover TF binding in rare cell types. In addition, DeepArk’s predictions may vary across different contexts, increasing the complexity of analysis, and this complexity can make analysis challenging but also generate novel hypotheses for mechanistic experiments. Thus, there’s still room for improvement in DeepArk. Novel CNN architectures and optimization of sequence lengths are expected to enhance regulatory activity prediction.

Over time, deep learning methods for identifying genomic functional elements are evolving and proliferating, thus provide robust support for our in-depth understanding of genome function and regulation mechanisms.

#### Identifying sequence variation

Genomic mutations are the basis of genetic diversity, and various changes in DNA sequences (e.g. SNVs, insertions, repeats) significantly impact on an organism’s physiology and behavior. Deep learning methods help efficiently extract features of different types of variations and flexibly identify various genomic data types.

In order to learn the internal connections of sequences and efficiently identify mutation sites, Poplin et al. developed the DeepVariant [30], which transforms the problem of distinguishing different mutation types and lengths into an image classification problem. The seven channels of the image are used to represent different gene data expression information, and the variant model Inception-v3 network of CNN is used to effectively extract image features, transforming the problem of identifying mutations from an expert-driven, technology-specific-statistical modeling process to a more automated process for optimizing a universal model for data.

Many non-coding variations are associated with disease risk. Zhou et al. utilized whole-genome deep learning analysis to predict the specific regulatory effects and adverse impacts of gene mutations [84], demonstrating the involvement of non-coding gene mutations in synaptic transmission and neuron development. Basset et al*.* [42] attempted to link neural network components with biological significance and predicted pathogenic single-nucleotide polymorphisms (SNPs) using CNNs. However, it lacks the capability to *de novo* annotate large genomes. Thus, by profiling the genome and variations of individual patients, deep learning models help predict the most effective drug and its dosage. As genomic sequences significantly influence mutation rates and are closely related to various functional genomic features, it is speculated that DNN models can learn information related to mutation rates by learning from extensive nearby sequences, thus providing better mutation rate estimates. MuRaL, a recently developed framework [85], was constructed with two modules learning mutation information from proximal and distal sequence context. It employs different types of DNNs to extract features, generating high-quality mutation rate maps for humans and multiple species. However, the model demonstrates relatively poor mutation rate estimates in certain genomic regions, particularly those overlapping recent segmental duplications on human chromosome 8, indicating potential inaccuracies and limitations in coverage. Moreover, while the model addresses fine-scale germline mutation rates, it may have limited applicability for predicting mutation rates of other types, such as small insertions and deletions.

In contrast to simple SVs like SNPs, complex SVs involve changes in large genomic sequences, often with multiple breakpoints, and are frequently overlooked. Researchers have developed a deep learning-based multi-object recognition framework called SVision [86]. It represents sequence variations as denoised images depicting differences between the sequence and its corresponding fragment on the reference genome, characterizing complex SVs with different structures from the genome.

Genomic variations are often the result of errors during DNA replication, recombination processes, or external environmental pressures and experimental handling. The application of deep learning in the field of genomic variations not only aids in mutation detection and identification of variation types but also contributes to our understanding of the biological functions and mechanisms related to genomic variation.

In addition to the above aspects, deep learning has been applied to other genome annotation tasks, such as predicting methylation sites [87], annotating transcription factor binding sites [46], and extending to epigenomics, structural genomics, functional genomics and other fields. So far, deep learning models have shown significant promise in genome annotation, bringing renewed vigor to our research efforts.

## CONCLUSION

In summary, we have presented an overview of conventional methods for genome annotation, discussed the fundamental frameworks of CNNs and RNNs. With the advancements in artificial intelligence and deep learning models’ bioinformatics finds its applications in diverse facets in genomics, i.e. protein-coding regions, regulatory elements, sequence variations, functional annotation and more. It is evident that the capacity of deep learning to handle high-dimensional and heterogeneous data has significantly aided researchers in genome annotation, analysis and classification.

Although deep learning models outperform many traditional methods in genomic annotation, machine learning may perform better for small datasets. Additionally, deep learning requires hardware support and a large amount of parallelism [88], so deep learning algorithms are not always superior to machine learning algorithms. Furthermore, when integrating natural language processing models into genomic data, interpretability challenges are inevitably encountered. The ability of deep learning to extract features from high-dimensional and heterogeneous data is a double-edged sword. The highly non-linear structure and overparameterization of DNNs enable precise problem-solving and predictions. However, the opaque nature of feature extraction and decision-making processes from the data hinders the biological interpretability of omics data, The specific training details of the model are not known. At present, the main approach is to infer and evaluate the features and weights of the model by studying the relationship between input and output, and provide a feature importance score. Additionally, the choice of deep learning models poses a challenging step. CNN excel in capturing crucial attributes of omics data, RNN leverage contextual information from sequences and autoencoders uncover hidden data distributions. Therefore, users are required to have an acute criteria and understanding for selection of the appropriate model based on the research objectives.

Although the impact of deep learning in the field of genome annotation may not have revolutionized the field as dramatically as it has in protein structure prediction, we anticipate a brighter future as new models continue to emerge, and omics data to proliferate. Deep learning holds the potential to bring significant advancements to the realm of genome annotation.

**Key Points**

- The ability to characterize data and features learning for conventional genome annotation methods was limited.
- Deep learning model becomes a powerful tool for genome annotation with broad application prospects.
- Two basic frameworks of deep learning models for sequence processing include Convolutional Neural Networks (CNN), Recurrent Neural Networks (RNN).
- The application field of deep learning in genome annotation mainly includes five aspects.
